# Asylum Matters

**Published:** 1900-12-01

**Authors:** 


### ASYLUM MATTERS.

### MENTAL DISEASE IN GENERAL HOSPITALS.

Out-patients' departments for mental diseases have been established in connection with several county asylums: for example, the West Riding at Wakefield and the Northampton at Berry Wood; and now a similar adjunct is to be instituted at the Newcastle Infirmary. Here it is intended to treat such cases as are mentally afflicted, but not sufficiently pronounced as to warrant their removal to an asylum. Presumably, therefore, only incipient cases will be dealt with at the waiting room of the Newcastle Infirmary. The departure is a good one, provided it can be ensured that the right sort of patients will apply, and provided also that the consulting physician is an expert in mental diseases. An additional advantage is that the Newcastle Infirmary is a medical school, and students cannot be too early trained in the diagnosis of all varieties of insanity. Of course there must be considerable risk that the wrong class of patients will apply, such as hysterical women and hysterical men too Probably the department will be most useful in the treatment of the premonitory symptoms of recurrent insanity, and in the early diagnosis of melancholia and general paralysis of the insane, the latter having so often a medico-legal aspect attached to it. It is a pity that alienist physicians do not invent a better name than "mental disease."

